# Optical Detection and Virotherapy of Live Metastatic Tumor Cells in Body Fluids with Vaccinia Strains

**DOI:** 10.1371/journal.pone.0071105

**Published:** 2013-09-03

**Authors:** Huiqiang Wang, Nanhai G. Chen, Boris R. Minev, Martina Zimmermann, Richard J. Aguilar, Qian Zhang, Julia B. Sturm, Falko Fend, Yong A. Yu, Joseph Cappello, Ulrich M. Lauer, Aladar A. Szalay

**Affiliations:** 1 Genelux Corporation, San Diego Science Center, San Diego, California, United States of America; 2 Department of Radiation Medicine and Applied Sciences, Rebecca & John Moores Comprehensive Cancer Center, University of California, San Diego, California, United States of America; 3 UCSD Division of Neurosurgery, University of California, San Diego, California, United States of America; 4 Department of Gastroenterology and Hepatology, University Hospital, Tuebingen, Germany; 5 Institute of Pathology, University Hospital, Tuebingen, Germany; 6 Department of Biochemistry, Rudolf Virchow Center for Experimental Biomedicine, and Institute for Molecular Infection Biology, University of Wuerzburg, Wuerzburg, Germany; Cincinnati Childrens Hospital Medical Center, United States of America

## Abstract

Metastatic tumor cells in body fluids are important targets for treatment, and critical surrogate markers for evaluating cancer prognosis and therapeutic response. Here we report, for the first time, that live metastatic tumor cells in blood samples from mice bearing human tumor xenografts and in blood and cerebrospinal fluid samples from patients with cancer were successfully detected using a tumor cell-specific recombinant vaccinia virus (VACV). In contrast to the FDA-approved CellSearch system, VACV detects circulating tumor cells (CTCs) in a cancer biomarker-independent manner, thus, free of any bias related to the use of antibodies, and can be potentially a universal system for detection of live CTCs of any tumor type, not limited to CTCs of epithelial origin. Furthermore, we demonstrate for the first time that VACV was effective in preventing and reducing circulating tumor cells in mice bearing human tumor xenografts. Importantly, a single intra-peritoneal delivery of VACV resulted in a dramatic decline in the number of tumor cells in the ascitic fluid from a patient with gastric cancer. Taken together, these results suggest VACV to be a useful tool for quantitative detection of live tumor cells in liquid biopsies as well as a potentially effective treatment for reducing or eliminating live tumor cells in body fluids of patients with metastatic disease.

## Introduction

Most cancer deaths result from the metastatic spread of cancer in which tumor cells escape from the primary tumor, relocate to distant sites and initiate new tumors [1]. Metastatic tumor cells found in body fluids such as blood, lymphatic, cerebrospinal and ascitic fluids have become important biomarkers in evaluating cancer prognosis and for monitoring therapeutic response. Importantly, prevention and elimination of such metastatic tumor cells may result in a significant reduction in morbidity and mortality.

Metastatic tumor cells in the peripheral blood, referred to as circulating tumor cells (CTCs), have been shown to be a prognostic biomarker for several types of solid tumors, including non-small cell lung cancer, breast cancer, colorectal cancer and prostate cancer [2]–[3]. Different methods have been investigated for the detection of CTCs [3]. Only the immunomagnetic technique-based platform CellSearch® (Veridex, Warren, NJ, USA) has been approved by the US Food and Drug Administration (FDA) for the detection of CTCs in breast, colon and prostate cancer patients [2]. However, detection by antibody-based techniques can present significant uncertainty due to the heterogeneity of antigen expression of CTCs. Hence, there is an urgent need for the development of a more comprehensive, sensitive, and cancer cell-specific method for CTC detection in order to improve the utility and potential benefit of CTC analysis in the clinic [3].

The spread of metastatic tumor cells to the cerebrospinal fluid (CSF) and leptomeninges results in leptomeningeal metastases (LM). The incidence of LM in cancer patients ranges between 5 and 15% and likely is on the rise as the survival of cancer patients increases. LM might also be underdiagnosed since some metastases may remain asymptomatic [4]. The prognosis for patients with LM is extremely poor with the median survival measured in months [5]. Treatment of LM is mainly palliative. Early diagnosis and effective treatment are critical to prevent important neurological deficits, improve quality of life and prolong survival. Methods for the diagnosis of LM include clinical examination, neuroimaging, and CSF analysis. Currently, the leading diagnostic laboratory test for LM is cytological examination of the CSF, a method with limited sensitivity and specificity.

Peritoneal carcinomatosis (PC) is the locoregional progression of cancers, being mostly of gastrointestinal and gynecological origins. At the time of diagnosis, about 10 to 15% of patients with gastrointestinal and gynecological cancers have already developed PC, a terminal condition and a consequence of the underlying systemic nature of the disease [6]. Treatment with cytoreductive surgery, followed by hyperthermic intraperitoneal chemotherapy has demonstrated a significant survival benefit [7]. This treatment, however, is expensive and is associated with a very high postoperative morbidity rate, ranging from 25 to 56% [6].

Recombinant vaccinia viruses (VACVs) carrying imaging genes have emerged as potential combined therapeutic and diagnostic agents (theranostics) due to their oncolytic potential and tumor specificity [8]. Using this VACV technology in combination with a cytospin slide-based system, we developed a simple, sensitive, specific, and widely applicable assay to successfully detect and enumerate live tumor cells in both preclinical and clinical liquid biopsies in an epithelial biomarker-independent manner. Unlike the FDA approved CellSearch® system, which relies on antibody-based detection of cell surface antigens of CTCs, VACV detection requires infection and expression of the virus-encoded marker protein by the target cell, thus only live CTCs are detected. Furthermore, we demonstrated that VACV infected and reduced live metastatic tumor cells both in the blood of mice bearing metastatic prostate cancer xenografts and in the ascitic fluid of a cancer patient with peritoneal carcinomatosis.

## Materials and Methods

### Cell Lines

The human prostate cancer cell line PC-3 and the human lung carcinoma cell line A549 were purchased from the American Type Culture Collection. The human prostate cancer cell line PC-3-RFP was generated as described previously [9]. HT-29 and A549 cells were cultured in RPMI 1640 (Mediatech, Manassas, VA, USA) supplemented with 10% FBS (Mediatech, Manassas, VA, USA). PC-3 cells were cultured in DMEM (Mediatech, Manassas, VA, USA) supplemented with 10% FBS. PC-3RFP cells were cultured in DMEM-supplemented with 10% FBS and 10 µg/ml of blasticidin. Cells were maintained at 37°C in a humidified atmosphere containing 5% CO_2_.

### Viruses

The cDNA encoding for TurboFP635 was PCR-amplified using the plasmid FUKW (kindly provided by Dr. Marco J. Herold, University of Wurzburg) as a template with primers FUKW-5 (5′-GTCGAC (*Sal* I) CACCATGGTGGGTGAGGATAGCGTGC-3′) and FUKW-3 (5′-TTAATTAA (*Pac* I) TCAGCTGTGCCCCAGTTTGC-3′). The PCR product was gel-purified, and cloned into the pCR-Blunt II-TOPO vector using Zero Blunt TOPO PCR Cloning Kit (life Technologies, Carlsbad, CA, USA). The resulting construct pCRII-FUKW was sequence confirmed. The TurboFP635 cDNA was then released from pCRII-FUKW with *Sal* I and *Pac* I, and subcloned into the VACV *A56R* (hemagglutinin, HA) shuttle vector HA-SL-hNET1 with the same cuts to replace the hNET cDNA. The resulting construct HA-SL-FUKW2 was sequence confirmed, and used to generate the recombinant virus GLV-1h254 using GLV-1h71 as the parental virus. GLV-1h68 and GLV-1h71 were described previously [10]–[11]. A schematic overview of the VACV strains used in this study is shown in Figure S1.

### Mouse Tumor Xenograft Models

This study was carried out in strict accordance with the recommendations in the Guide for the Care and Use of Laboratory Animals of the National Institutes of Health. The protocol of this study was approved by the Institutional Animal Care and Use Committee of Explora Biolabs (San Diego Science Center, San Diego, CA, USA; protocol number: EB11-025). Five- to six-week old nude mice (NCI:Hsd:Athymic Nude-Foxn1^nu^; Harlan, Indianapolis, IN, USA) were implanted subcutaneously with 5×10^6^ PC-3, PC-3RFP or A549 cells (in 100 µL PBS) on the right hind leg.

### Blood Sample Collection

Blood samples from healthy human donors were obtained from the San Diego Blood Bank (San Diego, CA, USA). Blood samples from patients with metastatic cancer were collected from the Pacific Oncology and Hematology Clinic (Encinitas, CA, USA), or Moores Cancer Center, the University of California, San Diego (La Jolla, CA, USA). All blood donors gave their written informed consent for study inclusion and were enrolled using the protocols approved by the institutional review boards of the Pacific Oncology and Hematology Clinic, and the University of California, San Diego, respectively. The blood was collected into EDTA tubes (BD Bioscience, San Jose, CA, USA). For the VACV-cytospin assay and CellSearch® system comparison study, one 7.5 mL whole blood sample from each patient was collected into a CellSave tube (Veridex, Raritan, NJ, USA) and shipped from the Pacific Oncology and Hematology Clinic to the Genoptix Laboratory (Carlsbad, CA, USA). Another 7.5 mL blood sample of the same draw was collected into EDTA tubes and shipped to Genelux Corporation (San Diego, CA, USA). To collect mouse blood samples, mice were anesthetized with 1% to 1.5% isoflurane and the blood was collected from the left ventricle of the heart into EDTA tubes using a 26-G needle (BD Bioscience, San Jose, CA, USA).

### Cell Spiking

PC-3 cells were labeled with the green fluorescence dye PKH67 using PKH67 Green Fluorescent Cell Linker Kit for General Cell Membrane Labeling (Sigma-Aldrich, St. Louis, MO, USA). 30∼60 single cells were spiked into 100 µL healthy mouse blood samples or 1 mL healthy human blood samples in triplicate. Blood samples from six healthy human donors and six healthy mice were tested.

### Red Blood Cell Lysis

Mononuclear cells and circulating tumor cells were enriched from whole blood samples by removing red blood cells with 1× RBC lysis buffer (eBioscience, San Diego, CA, USA) according to the manufacturer’s instruction.

### VACV Infection

The GLV-1h254 virus stock was diluted in DMEM supplemented with 2% FBS to yield a concentration of 2×10^7^ plaque-forming units (pfu)/mL. Nucleated cells from 1 mL of the whole blood following red blood cell lysis were resuspended in 0.5 mL of the diluted virus and incubated at 37°C for 24 h.

### Immunofluorescence Staining and Cell Deposition

Immunofluorescence staining procedures were carried out according to the manufacturer’s instructions. After staining, cells were washed once with 1× DPBS and resuspended in 1 mL of 1× DPBS. Hettich cytospin chambers (Hettich, Germany) were assembled and the stained cell suspension was directly added into cytospin reservoirs using a funnel card that creates cytospins with a diameter of 8.7 mm. The cells were deposited onto clean glass grid slides (VWR, West Chester, PA, USA) by centrifugation for 5 minutes at 1,500 rpm using a Hettich Universal 16 centrifuge (Hettich, Germany). Slides were dried for 10 minutes at room temperature. The 4′, 6-diamidino-2-phenylindole (DAPI) HardSet mounting medium (Vector Laboratories, Burlingame, CA, USA) was used for cell nuclei staining.

### Visualization and Enumeration of CTCs

An Olympus IX71 inverted epifluorescence microscope with PictureFrame® software was used to image cells on grid slides. The grids of each slide were checked for CTCs under the microscope one grid at a time. Infected CTCs showed very strong TurboFP635 expression together with staining positive for epithelial cell adhesion molecule (EpCAM), pan-cytokeratin (CK) and DAPI, but negative for CD45 (thus not leukocytes), and met the morphologic characteristics consistent with malignant cells, including large cellular size, high nuclear to cytoplasmic ratio, and visible nucleoli, were scored as CTCs.

### Detection of CTCs by the CellSearch® System

Samples were analyzed as previously described [12]–[13].

### Collection and Processing of CSF Samples

CSF samples from human patient donors were collected at the Moores Cancer Center, University of California, San Diego (La Jolla, CA, USA). All enrolled patients gave their written informed consent for study inclusion and were enrolled using the protocol approved by the institutional review board of the University of California, San Diego. Three to five mL CSF from each patient was collected. CSF samples were concentrated by centrifugation at 300× *g* for 5 minutes at room temperature. VACV infection and immunofluorescene staining were performed as described for CTCs above. Cells on the slide were imaged and enumerated under an epifluorescence microscope.

### Prevention and Therapy of CTCs

Groups of 8 mice bearing PC-3RFP tumor xenografts were treated with a single dose of 5×10^6^ pfu of GLV-1h68 (in 100 µL of PBS) either at 4 weeks (early treatment) or 7 weeks (late treatment) after tumor cell implantation. Mice treated with PBS at 4 weeks after implantation were used as controls. Mice were monitored weekly for CTCs using Clearbridge BioMedics CTC0 Capture System Prototype [14]. 100 µL of blood were drawn from mice through heart puncture and 80 µL were run through the biochip. At death or at the end of the experiment, all mice were dissected and examined for enlarged and/or fluorescing lumbar renal lymph nodes. The images were acquired using a Leica MZ 16 FA stereo-fluorescence microscope equipped with a FireWire DFC/IC monochrome CCD camera (Leica, Wetzlar, Germany). Digital images were processed with Photoshop 7.0 (Adobe Systems, Mountain View, USA).

### Treatment of a Patient with Peritoneal Carcinomatosis with GL-ONC1

Ascites from a patient with PC from gastric cancer, intraperitoneally treated with GL-ONC1 (clinical version of GLV-1h68), were analyzed. First, the concentration of cells in the ascitic fluid was determined. Then, the cells were collected by centrifugation, followed by fixation of the cell pellet in formalin (4%, Fischer, Germany) to a final concentration of 1×10^6^ cells/mL. After a repeated centrifugation of this cell suspension, the supernatant was discarded and the cell pellet was resuspended in a few drops of the remaining supernatant. This suspension was collected and mixed with hot agar (1% agarose), cooled, placed into a histology cassette and fixed again in formalin (4%). The cassette was then embedded in paraffin. From the resulting cell blocks, sections of 4 µm thickness were cut, deparaffinised and rehydrated by passages through xylene and graded alcohol and finally stained with haematoxylin and eosin for morphologic evaluation. IHC staining was performed using an automated immunohistochemistry staining system (VENTANA Benchmark; Ventana Medical Systems, Tucson, AZ, USA), using reagents from VENTANA according to the manufacturer’s protocol. Shortly, the slides were incubated with primary antibodies VACV-A27L (Genelux, CA, USA); Ber-EP4 (Dako, Germany) and visualized using iView DAB detection kit (Ventana) with horseradish peroxidase and DAB as chromogen. After DAB staining, slides were counterstained with haematoxylin, washed, dehydrated in a graded alcohol series and mounted with Cytosil (FISCHER Scientific, Germany). This study was approved by the Paul-Ehrlich-Institut, Germany. The trial was registered on http://www.clinicaltrials.gov (number NCT01443260). Written, informed consent was obtained from the patient.

### Statistical Analysis

Statistical analyses were performed with GraphPad Prism, version 5.03 (GraphPad Software Inc., San Diego, CA). Statistical analysis of survival was assessed using the log-rank test. Values of *P* less than 0.05 were considered significant.

## Results

### Optimization and Evaluation of VACV-cytospin CTC Detection Assay

In pilot experiments, the optimal viral dose for infection of tumor cells in blood samples using TurboFP635-expressing VACV, GLV-1h254, was determined to be 10^7^ pfu per mL of whole blood (Figure S2A). Using this dose, the capture efficiency, detection efficiency, and specificity as well as infection efficiency of the VACV-cytospin based CTC assay were evaluated. Thirty to 60 PC-3 human prostate cancer cells labeled with PKH67 (a green fluorescent dye) were spiked into 1 mL of whole blood from healthy human donors or 100 µL whole blood from healthy mice. The spiked whole blood samples were subjected to red blood cell lysis and VACV infection, followed by cytospin deposition. Each experiment was performed in triplicate and six individual human and mouse blood samples were used. The assay yielded similar results with both the human and mouse blood samples (Table S1). More than 70% of spiked tumor cells were captured on cytospin slides (70% capture efficiency), and more than 92% of these cells were infected by the virus (92% infection efficiency, for an overall detection efficiency of 65%). 100% of the infected cells identified on the slides were spiked tumor cells (100% detection specificity). In the spiking experiments, PC-3 cells were labeled with the PKH67 fluorescence dye and were washed before being spiked into blood samples. Spiked blood samples were then subject to RBC lysis and centrifugation before infection. All of these manipulations might adversely affect the viability of cells. Thus, not all spiked cells may have been alive at the time of infection, which would explain why we only achieved 92% infection efficiency, but not 100%.

To further demonstrate that GLV-1h254 specifically infected only spiked tumor cells, but not healthy blood cells, both human and mouse whole blood samples with or without spiked PC-3 cells were infected with GLV-1h254 and then stained with anti-human or anti-mouse CD45 monoclonal antibodies to identify leukocytes. All cells showing high-level expression of TurboFP635 (thus infected with GLV-1h254) were CD45-negative, whereas all cells staining positive for CD45 showed no TurboFP635 expression (Figure S2B).

### Detection and Identification of Live Human CTCs in Blood Samples from Mice Bearing Human Tumor Xenografts

**Figure 1:**
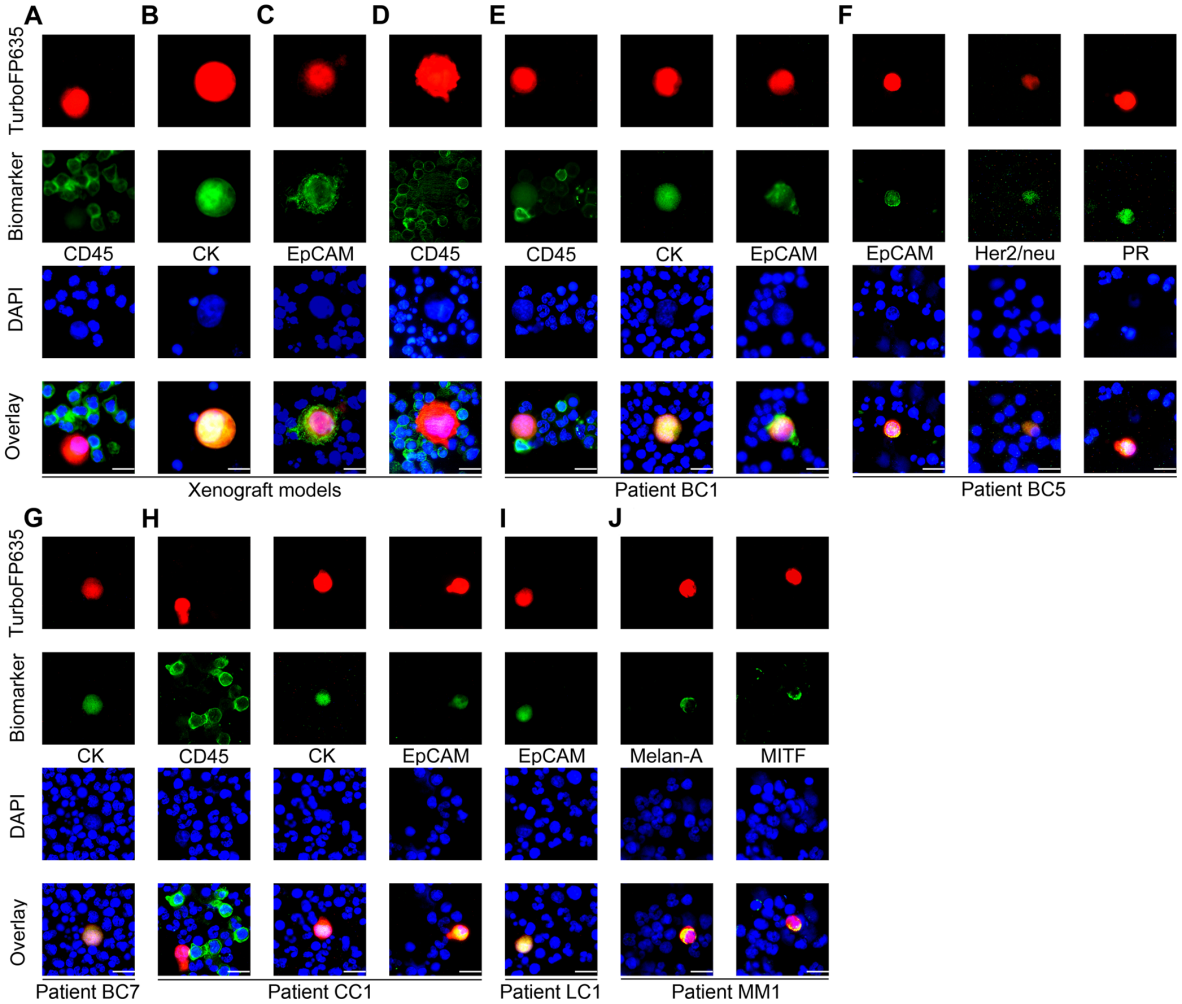
Detection and identification of live human CTCs in blood samples. (A) VACV GLV-1h254 detected live CTCs in mice bearing human PC-3 prostate cancer xenografts. Infected CTCs were CK^+^ (B) or EpCAM^+^ (C). Live CTCs were also detected by GLV-1h254 in mice bearing human A549 non-small lung cancer xenografts (D). (E) The live CTCs in the patient BC1 were identified as TurboFP635^+^/EpCAM^+^ or CK^+^/CD45^−/^DAPI^+^ cells. (F) The live CTCs detected in the patient BC5 were confirmed to express EpCAM, PR, or HER2/neu markers. (G) The live CTCs in the patient BC7 were confirmed as TurboFP635^+^/CK^+^/DAPI^+^ cells. (H) The live CTCs in the patient CC1 with metastatic colorectal cancer were identified as TurboFP635^+^/EpCAM^+^ or CK^+^/CD45^−/^DAPI^+^ cells. (I) The live CTCs in the metastatic lung cancer patient LC1 were confirmed as TurboFP635^+^/EpCAM^+^/DAPI^+^ cells. (J) The VACV detected live CTCs in the metastatic melanoma patient MM1 were shown to express melanoma tumor markers Melan-A or microphthalmia-associated transcription factor.

The mouse xenograft model of human prostate cancer was generated by implanting 5×10^6^ PC-3 tumor cells into the right hind leg of nude mice. 100 µL of whole blood samples were taken from these mice by cardiac puncture for CTC analysis using the VACV-cytospin assay. Infection of blood samples with GLV-1h254 *ex vivo* revealed microscopically that infected cells were much larger than surrounding CD45^+^ immune cells, displayed bright TurboFP635 fluorescent signal, contained nuclei, and were CD45^−^ (Figure 1A). These infected cells were also CK^+^ (Figure 1B) or EpCAM^+^ (Figure 1C), indicating that the infected cells were of epithelial origin, as expected for PC-3-derived CTCs. In another experiment, CTCs were also detected and identified as TurboFP635^+^/CD45^−/^DAPI^+^ cells in mice bearing late-stage human A549 non-small cell lung cancer xenografts (Figure 1D).

### Detection and Identification of Live CTCs in Blood Samples from Patients with Breast Cancer

Whole blood samples from seven patients with breast cancer were analyzed using the VACV-cytospin assay (Table S2). The volume of each sample varied from 5 to 15 mL. Live CTCs were detected in three patients. No CTCs were found in the remaining four patients (4–7 mL). To confirm the absence of CTCs in these patients, the same blood samples were analyzed using immunostaining for CK and EpCAM. Again, no CTCs were detected.

Patient BC1 had stage III breast cancer with histological diagnosis of estrogen receptor (ER)- negative, progesterone receptor (PR)-negative and human epidermal growth factor receptor 2 (HER2/neu)–negative, suggesting an aggressive disease, and had been undergoing chemotherapy and irradiation treatments before the blood sample was drawn. The VACV-cytospin assay detected a total of 66 live CTCs in a 5.5 mL whole blood sample. The CTCs identified by GLV-1h254 (TurboFP635^+^/CD45^−/^DAPI^+^) were also CK^+^ or EpCAM^+^ (Figure 1E). Another patient, BC5, had stage I breast cancer with histological diagnosis of ER^-^, PR^+^ and HER2/neu^+^. Fifteen live CTCs were detected in a 5 mL blood sample from this patient. These live CTCs showed not only EpCAM expression, but also PR and HER2/neu expression (Figure 1F **)**, consistent with the histological diagnosis. Similar to patient BC1, patient BC7 also had stage III breast cancer with histological diagnosis of ER^-^, PR^-^ and HER2/neu^-^. The patient had been undergoing chemotherapy, irradiation and trastuzumab treatments before the blood sample was drawn. Only 3 live CTCs were found in a 3 mL blood sample. The CTCs were identified as TurboFP635^+^/CK^+^/DAPI^+^ cells (Figure 1G).

### Detection and Identification of Live CTCs in Blood Samples from Patients with Other Types of Cancer

We analyzed blood samples from patients with metastatic colorectal cancer, lung cancer and melanoma using the VACV-cytospin assay (Table S3). Patient CC1 with metastatic colorectal cancer had been undergoing chemotherapy and bevacizumab treatments before the whole blood sample was drawn. Forty-one live CTCs were detected in a 5 mL blood sample from this patient. These infected live CTCs were confirmed as TurboFP635^+^/EpCAM^+^ or CK^+^/CD45^−/^DAPI^+^ cells (Figure 1H). The lung cancer patient LC1 with brain metastases had not been undergoing any treatment. Fourteen live CTCs were identified in a 5 mL blood sample from this patient. These live CTCs also displayed EpCAM expression (Figure 1I). Twenty-four live CTCs were detected by GLV-1h254 in a 5 mL whole blood sample from the patient MM1 with malignant metastatic cutaneous melanoma and these CTCs were confirmed to express melanoma markers microphthalmia-associated transcription factor or Melan-A (Figure 1J).

### Side-by-side Comparison of VACV-cytospin Assay and CellSearch® System

Blood samples from 10 patients with metastatic breast cancer and colon cancer were evaluated for CTCs using the two CTC assays (Table S4). From each patient, one 7.5 mL blood sample was collected in a CellSave tube and shipped to Genoptix Laboratory (Carlsbad, CA) for CTC detection using the CellSearch® system. Another 7.5 mL blood sample from the same blood draw was collected in EDTA tubes and analyzed for CTCs using the VACV-cytospin assay. The live CTCs identified by VACV were confirmed with immunostaining as TurboFP635^+^/CK^+^/CD45^−/^DAPI^+^ cells. Both assays detected CTCs in patients 1–4. In addition, the VACV-cytospin assay detected 2 CTCs in patient 7, but no CTCs were detected in this patient by the CellSearch® system. Neither assay detected any CTCs in the remaining 5 patients (Table 1). The VACV-cytospin assay detected slightly more CTCs in patients 1 and 2, but a few less CTCs in patient 3 than the CellSearch® system. Interestingly, the CellSearch® system detected 103 CTCs in patient 4 while only 5 CTCs were identified using the VACV-cytospin assay, suggesting most of the CTCs in patient 4 might not be alive since the CellSearch® assay detects both live and dead CTCs whereas the VACV-cytospin assay only detects live CTCs. In summary, 4 out of 10 patients were positive for CTCs as determined by the CellSearch system. All these patients (100%) were also reported to be positive by the VACV-cytospin assay. Five of 6 patients (83%) determined to not have CTCs by the CellSearch system were confirmed to be negative for CTCs by the VACV-cytospin assay. Thus, the sensitivity of the VACV-cytospin assay to the CellSearch system was 100% with specificity of 83%, positive predictive value of 100%, and negative predictive value of 83%.

**Table 1:** Side-by-side comparison of VACV-Cytospin assay and CellSearch® system.

Patient ID	01	02	03	04	05	06	07	08	09	10
**CTC # (CellSearch®)**	7	27	5	103	0	0	0	0	0	**0**
**Live CTC # (VACV)**	10	35	2	5	0	0	2	0	0	**0**

Values indicate the number of CTCs per 7.5 mL of blood.

### Characterization of CTCs Identified in Blood Samples from Mice with Human Tumor Xenografts and Patients with Cancer

**Figure 2:**
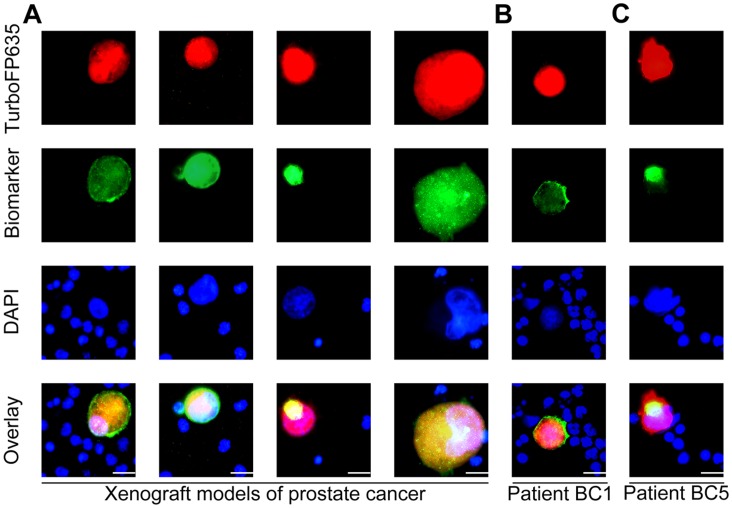
Characterization of CTCs in blood samples detected with GLV-1h254. (**A**) The CTCs in mice bearing human PC-3 prostate cancer xenografts showed high-level expression of CD44, ALDH1, vimentin and N-cadherin. (**B**) The CTCs from the human metastatic breast cancer patient BC1 showed the strong expression of CD44. (**C**) The live CTCs in the human metastatic breast cancer patient BC5 showed the strong expression of ALDH1.

Recent studies have indicated that CTCs may be linked to both cancer stem cells (CSCs) and the epithelial-mesenchymal transition (EMT) process [15]–[16]. To elucidate the relationship of CTCs with CSCs and EMT, we analyzed CTCs for the expression of CSC and EMT markers. The CTCs identified with GLV-1h254 in mice bearing human PC-3 prostate cancer xenografts displayed high levels of the expression of the CSC markers CD44 and aldehyde dehydrogenase 1 (ALDH1) as well as the EMT markers vimentin and N-cadherin (Figure 2A). Furthermore, the CTCs identified in the breast cancer patients BC1 and BC5 showed high-level expression of CD44 (Figure 2B) and ALDH1 (Figure 2C), respectively. The features of CSCs as well as phenotypic change characteristics of the EMT possessed by CTCs might allow them to disseminate effectively during the progress of cancer metastases, resulting in the formation of secondary tumors by extravasation and colonization in distant organs.

### Detection and Identification of Live Cancer Cells in CSF Samples from Patients with Cancer

**Figure 3:**
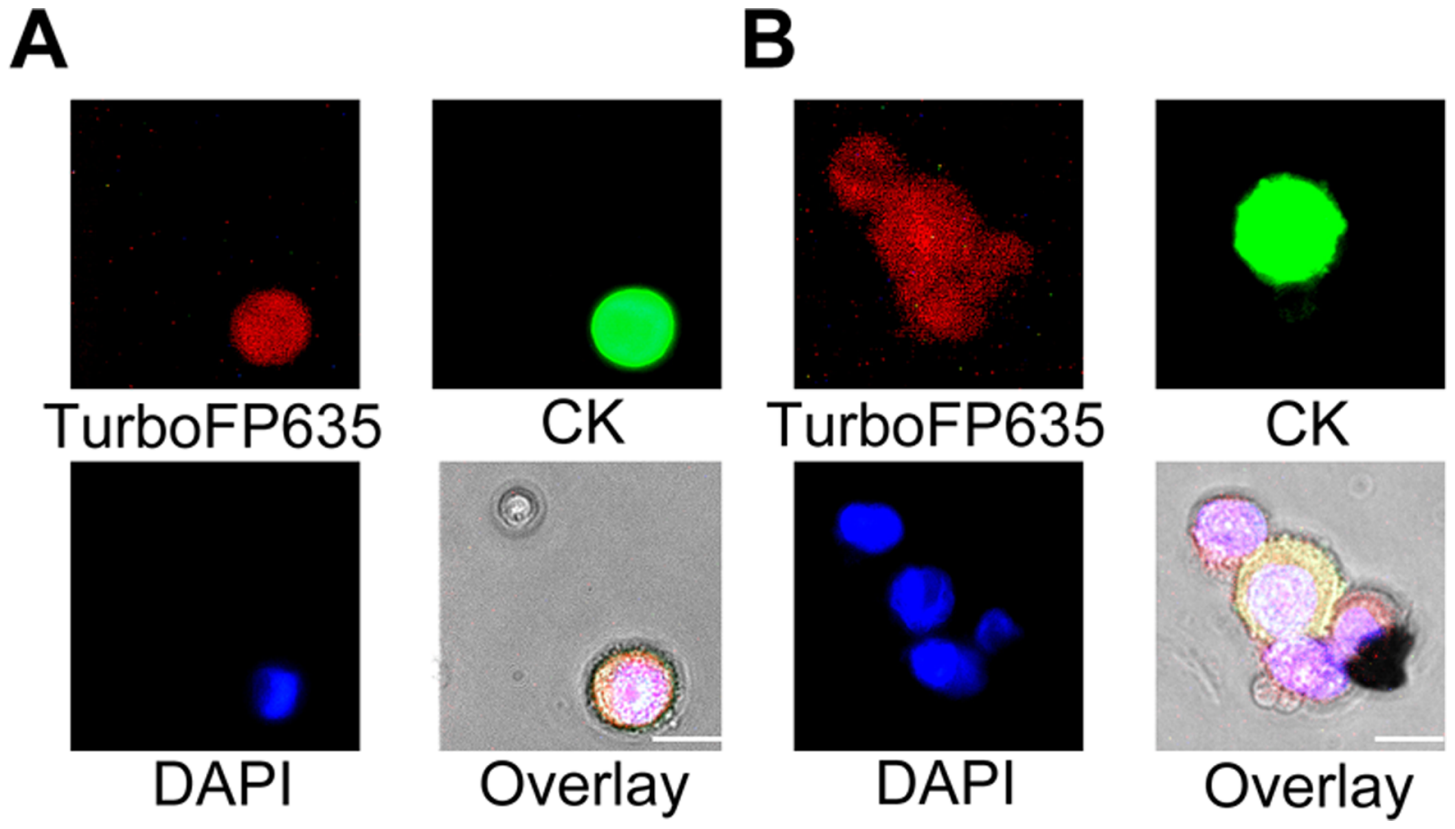
Detection and identification of live tumor cells in a CSF sample. (A) The live tumor cells in the CSF sample of a patient with metastatic breast cancer were identified as TurboFP635^+^/CK^+^/DAPI^+^ cells. (B) A live cancer cell cluster with heterogeneous expression of CK in the CSF sample was identified by VACV.

CSF samples from seven patients with glioblastoma multiforme, metastatic colorectal carcinoma, metastatic breast cancer and metastatic esophageal cancer were analyzed using the VACV-cytospin assay (Table S5). The volume of each sample ranged from 3 to 5 mL. A total of 23 TurboFP635^+^ cells with large nuclei were found in the 3 mL CSF sample from the patient CSF7 with metastatic breast cancer (Figure 3). Among these, 16 cells showed high-level expression of CK (Figure 3A) and the rest of the infected cells showed very low level or no expression of CK (Figure 3B). No infected cells were found in the CSF samples from the other six patients. To confirm the absence of cancer cells in these six CSF samples, infected samples were further analyzed using immunostaining for CK. No CK^+^ cells were detected in these six patients that were negative for TurboFP635.

### Prevention and Therapy of CTCs in Mice Bearing Human Prostate Cancer Xenografts

Nude mice were implanted with the modified human prostate cancer cell line PC-3RFP, expressing red fluorescent protein (RFP) on the right hind leg to facilitate CTC detection using the ClearBridge biochip [14]. Mice were monitored for RFP-expressing CTCs weekly after tumor cell implantation. CTCs captured on the biochip were visualized and counted under a fluorescent microscope. Mice were treated with a single intravenous dose of 5×10^6^ pfu of GLV-1h68 (expressing GFP) either at 4 weeks (early treatment, n = 8) or at 7 weeks (late treatment, n = 8) after tumor cell implantation. Mice treated with PBS at 4 weeks after tumor cell implantation were used as controls (n = 7).

**Figure 4:**
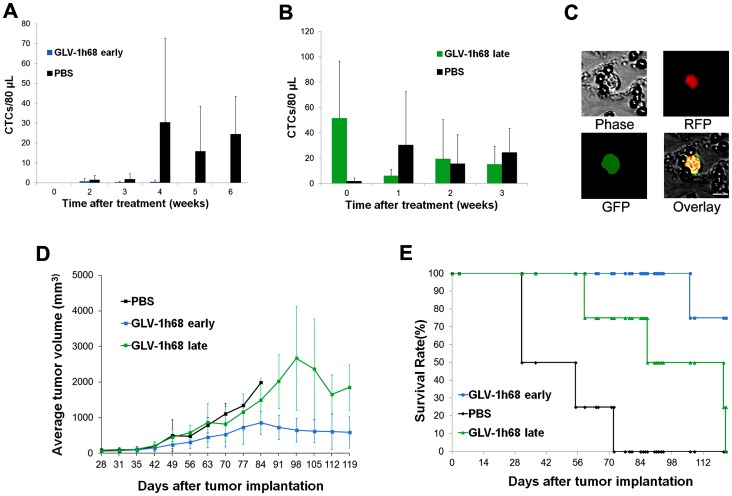
Prevention and therapy of CTCs in mice bearing human prostate cancer. (A) GLV-1h68 early treatment prevented CTC formation in the majority of mice (7/8) while mice in the PBS group showed an increase in CTC numbers. (B) GLV-1h68 late treatment resulted in a dramatic decrease in CTC numbers while CTC numbers in the PBS group increased greatly, and then fluctuated over time. (C) Almost all CTCs detected in the GLV-1h68 late treatment group were GFP-positive (infected) at two weeks after treatment. (D) Primary tumors regressed after GLV-1h68 early or late treatment. (E) Both GLV-1h68 early and late treatments significantly prolonged mouse survival (p = 0.002, GLV-1h68 early vs. PBS; p = 0.006 GLV-1h68 late vs. PBS).

No CTCs were detected in any of the mice through 4 weeks after tumor cell implantation (prior to any treatment). All mice in the PBS-treated control group were CTC positive at one or more time points starting at 5 weeks after tumor cell implantation. In contrast, only one animal in the GLV-1h68 early treatment group had any detectable CTC’s after virus treatment (3, 1, and 2 CTCs at 2, 3, and 4 weeks after treatment, respectively, Figure 4A). Thus, early treatment significantly reduced CTC formation in mice bearing human prostate cancer tumors.

Mice in the late treatment group had a significant number of CTCs before virus treatment, ranging from 12 to 103 CTCs per 80 µL of blood. A dramatic decrease in CTC numbers was observed one week after virus treatment (Figure 4B). Interestingly, at 1 week after treatment, 52.9% of CTCs were GFP positive, thus were infected by GLV-1h68. At 2 weeks after treatment, the number of CTCs remained at reduced levels, and almost all CTCs (99.6%) were GFP positive (Figure 4C).

While primary tumors kept growing in the PBS group, both early and late treatments with GLV-1h68 resulted in tumor regression (Figure 4D) and significantly prolonged survival in comparison with PBS treatment (Figure 4E). The average survival days were 126.3 and 97.3 days for the early and late treatment groups, respectively, versus 52.7 days for the PBS treatment group. At death or at the end of the experiment, all mice were dissected and metastases were examined under a fluorescent stereo microscope. All mice in the PBS group had detectable lumbar and renal lymph node metastases (Figure S3). In contrast, only one out of 8 mice in the early treatment group had a slightly enlarged lumbar lymph node, the other mice in this group had no detectable lumbar or renal lymph node metastases. Although all mice in the late treatment group had detectable lumbar and renal lymph node metastases, these metastases were smaller in size compared to the PBS group.

### Therapy of Metastatic Cancer Cells in the Ascites of a Patient with PC from Gastric Cancer

**Figure 5:**
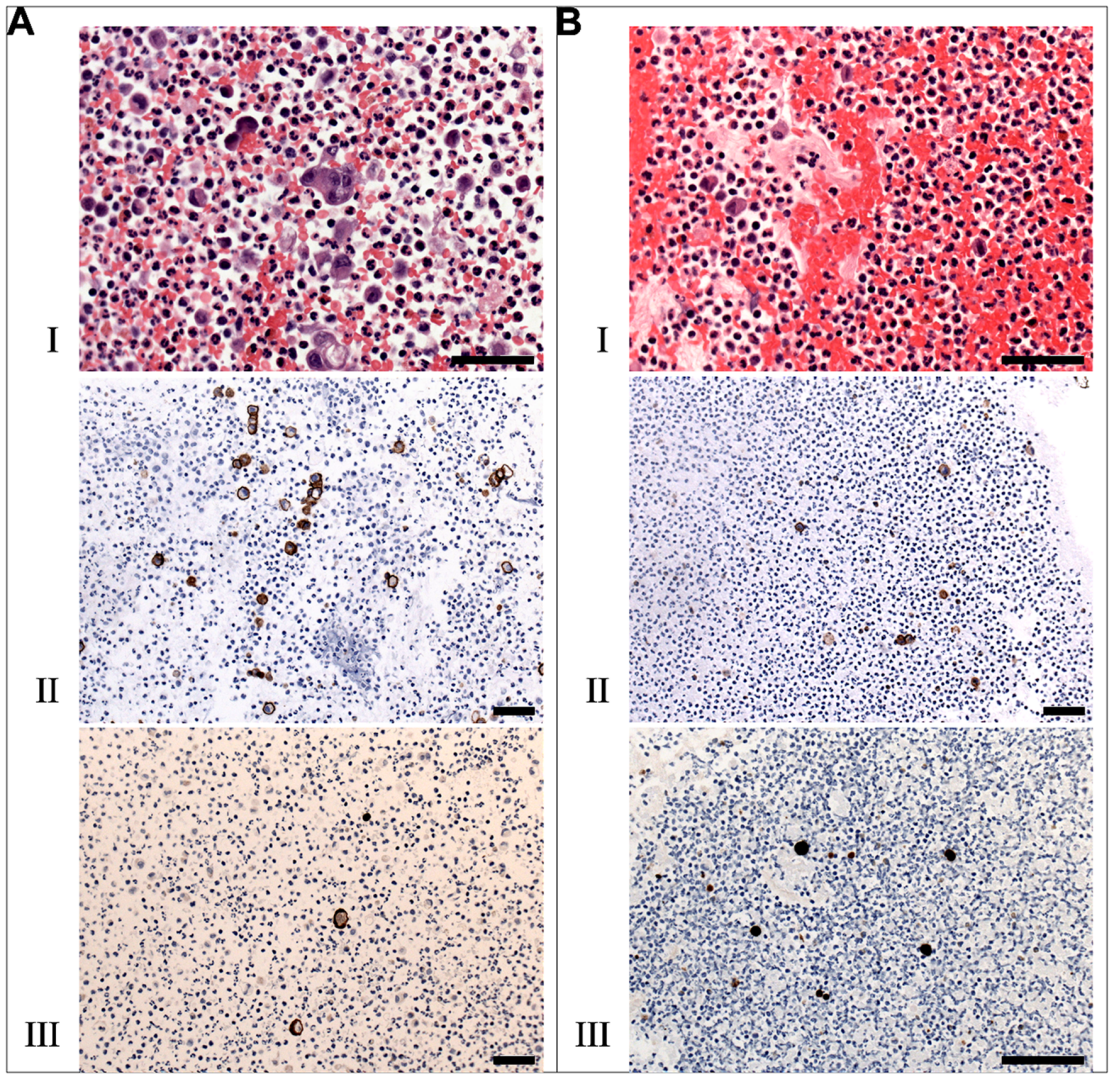
Therapy of cancer cells in the ascites of a cancer patient with peritoneal carcinomatosis. Cell block sections of malignant ascitic cells were stained with haematoxylin and eosin (A-I, B-I) at three (A) and seven (B) days after virus treatment. EpCAM positive cells in the malignant ascites sections were detected using an anti-EpCAM antibody (A-II, B-II). GL-ONC1 infection of single cells was detected using an anti-VACV-A27L antibody (A-III, B-III). Scale bars represent 50 µm.

Tumor cell-containing ascites from a patient with PC from gastric cancer were isolated three and seven days after the first intraperitoneal treatment with 10^7^ pfu of GL-ONC1 (clinical version of GLV-1h68). Ascitic cells were fixed in buffered formalin, and a cell block was made and paraffin embedded. Paraffin sections were stained with haematoxylin and eosin for morphologic evaluation. Using either anti-EpCAM or anti-vaccinia specific antibodies, around 5% of all cells were found to be EpCAM-positive three days after treatment, and only around 5–10% of these cancer cells were VACV positive at the same time point (Figure 5). The background population showed an evidence of significant acute inflammation with an increase in neutrophils. In contrast, four days later (i.e. 7 days after treatment), less than 2% of all ascitic cells were still EpCAM-positive, and more than 90% of these cancer cells were VACV positive. The VACV-positive cancer cells morphologically showed significant degenerative changes (in the process of cell death). These promising results suggest that GL-ONC1 can effectively remove live tumor cells in the ascites of patients with PC.

## Discussion

Metastatic tumor cells in body fluids such as blood, lymphatic fluids, CSF, and ascites represent a great challenge for cancer diagnosis and treatment. Cancer metastasis mediated by metastatic tumor cells is responsible for more than 90% of cancer patient deaths [17]. The ability to detect and treat metastatic tumor cells will have a great impact on reducing cancer-related morbidity and mortality. In the present study, we demonstrated the feasibility of using VACV to detect live metastatic tumor cells in blood from both mouse models and patients with cancer, as well as in the CSF sample from a patient with breast cancer. Importantly, VACV was shown to be effective in preventing and treating CTCs in the mouse model, and in reducing tumor cells in the ascites of a patient with PC from gastric cancer.

VACV preferentially infects and replicates in cancer cells [8], [18]. Cancer cells are fast growing cells that can provide ample nutrients, such as nucleotide pools, for VACV replication. In addition, cancer cells lack intact interferon signaling pathways and/or apoptotic pathways that limit virus replication in healthy cells. The tumor selectivity of VACV can be further increased mainly through inactivation of viral genes that are critical for efficient viral replication in healthy cells, but are dispensable upon infection of cancer cells [8]. VACV is known to infect various types of cancer cells. VACV enters cells through either plasma membrane fusion or macropinocytosis after attaching to cell surface glycosaminoglycans or extracellular matrix protein laminin [19]. The entry pathways are virus strain and cell type-dependent. Although several cellular proteins have been indicated to participate in VACV entry, their significance is not fully revealed. These proteins might be required for the specific VACV strains and cell types used. The cellular receptors shared by all poxviruses have yet to be identified. The use of redundant attachment modes and alternative entry pathways may contribute to the ability of VACV to infect a broad range of cancer cells. The recombinant vaccinia virus employed for the detection of metastatic tumor cells in this study was GLV-1h254 that expresses TurboFP635 (Katushka) under the control of the VACV strong synthetic late promoter for easy and sensitive detection of infected cells. TurboFP635 is a bright far-red fluorescent protein (seven to ten fold brighter than HcRed and mPlum), characterized by fast maturation and a high photo stability [20]. These features make it an ideal protein for visualization and detection of infected tumor cells. The vaccinia strong synthetic late promoter was intentionally selected to control the expression of TurboFP635, not only to achieve high-level expression of the protein, but also to avoid visualizing infected healthy blood cells. VACV is known to infect monocytes, B lymphocytes and natural killer cells in which infection is abortive, resulting in the expression of only early viral proteins, but not late proteins [21]. To evaluate the tumor cell specificity of GLV-1h254, whole blood samples from six healthy human and mouse donors were infected with GLV-1h254 after red blood cell lysis. No TurboFP635-expressing cells were found. Furthermore, when pre-labeled tumor cells were spiked into whole blood samples from healthy human and mouse donors, only spiked tumor cells showed strong TurboFP635 fluorescent signals. Importantly, GLV-1h254-infected cells found in whole blood samples from human cancer patients with metastatic breast cancer, colorectal cancer, lung cancer and melanoma and from mice bearing human lung and prostate tumors were confirmed to be CTCs by immunostaining for epithelial cell biomarkers, such as CK and EpCAM, or tumor-specific antigens. Thus, GLV-1h254 is highly tumor cell-specific for the detection of human CTCs in both patients with cancer and in mice bearing human cancer xenografts.

Most of the widely used CTC assays are based on the detection of epithelial cell markers such as CK and/or EpCAM [2]. Theoretically, these assays can detect both cancerous and healthy epithelial cells circulating in the peripheral blood and, therefore, are not strictly cancer-specific. Additionally, down-regulation of EpCAM and CK was seen for CTCs in the peripheral blood [22]–[25]. Thus, CTC detection assays using EpCAM and/or CK as biomarkers, such as the FDA-approved CellSearch® system, may only detect CTCs with high-level expression of EpCAM and/or CK while CTCs with low-level expression of these biomarkers or CTCs that do not express these biomarkers will be left undetected. Furthermore, the performance of antibody-based assays relies on the quality of antibodies and pre-validation of each antibody is required. On the contrary, infection of CTCs with VACV is independent of the expression of biomarkers, such as EpCAM and CK, thus, detection of CTCs with VACV eliminates bias related to the use of antibodies, and VACV can be potentially used to detect CTCs of any tumor type, not limited to CTCs of epithelial origin.

The VACV-cytospin assay only detects live CTCs, whereas CellSearch® system identifies CTCs irrespective of their viability. The performance of the CellSearch system has been previously evaluated [13]. In a side-by-side comparison of the VACV-cytospin assay and the CellSearch® system, blood samples from 10 patients with breast or colon cancer were tested. CTCs were enumerated independently by the two methods. Both methods detected CTCs in patients 1 to 4, but not in patients 5, 6, 8, 9 and 10. In addition, the VACV-cytospin assay detected 2 CTCs in patient 7, but no CTCs were detected in this patient by the CellSearch® system. In patients 1 and 2, the VACV-cytospin assay detected a slightly higher number of CTCs than did the CellSearch® system, suggesting that detection efficiency of the VACV-cytospin assay was comparable to or slightly higher than that of the CellSearch® system. In patient 3, a slightly lower number of CTCs were detected by the VACV-cytospin assay as compared to the CellSearch® system. Intriguingly, in patient 4, 103 CTCs were enumerated by the CellSearch® system while only 5 CTCs were found by the VACV-cytospin assay in a 7.5 mL blood sample from the same blood draw, indicating that most of CTCs in the patient 4 might have been dead. The timing of cell death in this case is not apparent from this analysis. It is possible, however unlikely, that the CTCs in this patient were killed after obtaining the blood sample, although appreciable numbers of non-live cells were not found in any other patient samples and all samples were handled and processed in the same manner. Alternatively, non-live CTCs may be prevalent in the circulation of some cancer patients. Use of the VACV-cytospin assay in combination with the CellSearch® assay could offer a tool to investigate the frequency and possible significance of this occurrence. Live CTCs detected by the VACV-cytospin assay might be more clinically relevant than total CTCs identified by the CellSearch® system since only live CTCs can potentially initiate new metastases.

Cancer metastasis is a comprehensive process that involves CSCs, EMT, and CTCs [26]. It seems that CTCs and CSCs are not necessarily separate populations of cancer cells, as CTCs generated in the process of EMT can bear features characteristic of CSCs [27]. We characterized VACV-identified live CTCs by immunostaining for CSC markers CD44 and ALDH1 as well as EMT markers vimentin and N-cadherin. It was found that VACV-identified CTCs co-expressing these markers, suggesting a critical role of these CTCs in the formation of cancer metastases.

In addition to the detection of CTCs in blood samples, we were also able to identify a significant number of metastatic tumor cells in the CSF sample from a patient with late-stage metastatic breast cancer. Our method can be an important addition to the CSF analysis currently used in the clinic for the diagnosis of LM [4]. It is conceivable that the VACV-cytospin assay can be also widely applied to the detection of metastatic tumor cells in other liquid biopsies, such as lymphatic, plural, and peritoneal fluids, and even in bone marrow samples.

 Oncolytic adenovirus and herpes simplex virus-1 expressing GFP have also been developed for the identification of cancer cells in liquid biopsies [28]–[31]. Fong *et al.* reported a proof-of-principle study demonstrating that a GFP-expressing oncolytic herpes simplex virus (HSV), NV1066 was able to identify human cancer cells spiked into samples of whole blood through detection of virus-mediated GFP expression in infected cancer cells by fluorescence microscopy and flow cytometry [31]. Infected cancer cells were then isolated by fluorescence-assisted cell sorting for subsequent molecular characterization. This method allowed detection of as little as 10 cancer cells in 10 mL whole blood. This was the first study showing the utility of oncolytic viruses for detection of rare cancer cells in blood samples. A follow-up study from the same lab showed that cancer cells in pleural fluid samples from two cancer patients could be detected by the same virus, demonstrating the potential clinical use of this technique [29]. Its applicability, however, has not been validated with blood samples from cancer patients. Furthermore, this assay provides only qualitative information about the presence or absence of cancer cells in liquid biopsy samples. The use of this assay for quantitative determination of cancer cells needs to be further investigated. In contrast, we showed that our method was able to quantitatively enumerate cancer cells in both blood and CSF samples from patients with various cancers as well as in blood samples from mice bearing human tumor xenografts. In addition to HSV, Kojima *et al*. reported that a telomerase-specific replication-selective adenovirus expressing GFP, OBP-401, successfully enumerated CTCs in blood samples from patients with various types of cancer [30]. When the OBP-401 assay was compared with the CellSearch system in detecting CTCs in both early breast cancer patients and metastatic breast cancer patients, a great discrepancy in patients with CTCs between both assays were noticed although the CTC detection rates for the two assays were comparable [28]. Both assays detected CTCs in 15% of the patients with early breast cancer. None of the patients, however, were positive for both methods. Among the patients with metastatic breast cancer, only 18% of the patients were identified as positive by both approaches while 24% and 36% of the patients were identified solely as positive by the OBP-401 assay or the CellSearch assay, respectively. In contrast, the VACV-cytospin assay showed a great consistency with the CellSearch system in patients with CTCs. Four and 5 out of 10 patients were determined to be positive and negative for CTCs, respectively, by both assays. Only 1 out 10 patients was positive for the VACV-cytospin assay alone, and negative for the CellSearch system. The OBP-401 and CellSearch assays might detect different populations of CTCs in the blood. The CellSearch detects CK^+^ and EpCAM^+^ cells whereas the OBP-401 assay identifies cells that express the coxsackie adenovirus receptor and telomerase. Due to the heterogeneous expression of the coxsackie adenovirus receptor on tumor cells [32], the wide applicability of this virus needs to be further investigated. Further, hematopoietic stem cells in the peripheral blood express high-level telomerase, which may cause false-positive results.

 GLV-1h68, possibly one of the best-studied oncolytic vaccinia viruses, is highly tumor-specific and effective in treating many types of human and canine tumors in preclinical models [8], [33]. GLV-1h68 has been tested in a phase I clinical trial and is being tested in more phase I and II trials, demonstrating a safe profile and initial evidence of anti-tumor activity in cancer patients [34]. Previously, we have shown that treatment with GLV-1h68 resulted in tumor regression and reduced lymphatic metastases in mice bearing human prostate cancer PC3 xenografts [35]. We have further shown that GLV-1h68 preferentially colonizes metastases compared to primary tumors [9]. In this study, we sought to investigate how GLV-1h68 treatment can affect CTC formation and its kinetic change in the same tumor model. To mimic the clinical situation in patients with early or late stage prostate cancer, mice bearing human prostate cancer PC3 xenografts were treated with a single intravenous dose of GLV-1h68 either before or after CTCs were detected. Surprisingly, GLV-1h68 early treatment completely prevented CTC formation in 7 out of 8 treated mice. Probably, early treatment inhibited migration of tumor cells from primary tumors since no lymph node metastases were detected in these mice. GLV-1h68 late treatment reduced the number of CTCs, and more than 50% and nearly 100% of CTCs captured were infected by GLV-1h68 (GFP positive) at 1 week and at 2 weeks after treatment, respectively. Smaller and fewer lymph node metastasis were seen in the late treatment group compared to the PBS control group. Both early and late treatments with GLV-1h68 resulted in the regression of primary tumors and significantly prolonged survival in comparison with PBS treatment. In our study, the absence and decreased number of CTCs were consistent with absent or reduced metastases, supporting the current hypothesis that in metastatic prostate cancer, the level of CTCs is strongly related with lymphatic metastases and overall survival [2].

PC is generally regarded as a fatal disease. In a proof-of-principle study, a patient with PC from gastric cancer was treated with a single low dose of GL-ONC1 delivered intraperitoneally. Interestingly, we found that more than 90% of cancer cells in the ascites of this patient were infected just 7 days after treatment, indicating that GL-ONC1 replicates efficiently *in situ* in the peritoneal cavity, a very promising result for the possible clearance of such malignant cells from the peritoneal cavity in patients with PC.

To our knowledge, this is the first report demonstrating the feasibility of using VACV to detect live metastatic tumor cells in liquid biopsies, and to treat these malignant cells in body fluids in both mouse models and patients with cancer. The VACV-cytospin assay can potentially be a widely applicable method to detect live metastatic tumor cells of any tumor type, not limited to those of epithelial origin. Identification of live metastatic tumor cells in liquid biopsies may provide valuable diagnostic and prognostic information specific for each individual patient. Prevention and elimination of metastatic tumor cells with VACV would result in great reductions in morbidity and mortality.

## Supporting Information

Figure S1 **Schematic representation of the genomic structures of the recombinant VACVs GLV-1h68 and GLV-1h254.** PE/L, PL, P11, and P7.5 are VACV synthetic early/late, synthetic late, 11K, and 7.5K promoters, respectively. TfR is a human transferin receptor cDNA inserted in the reverse orientation with respect to the promoter PE/L.
(TIF)

Figure S2 **Characterization of VACV-cytospin assay.** (A) Infection efficiency of PC-3 cells spiked in the mouse and human blood at escalated doses of virus. (B) GLV-1h254 infected only PC-3 cells spiked in the whole human blood, but not immune cells (red: TurboFP635; green: CD45; blue: nuclei).
(TIF)

Figure S3 **The effect of GLV-1h68 on lymphatic metastases in mice bearing human PC3 prostate cancer xenografts.** All mice in the PBS group had detectable lumbar and renal lymph node metastases. In contrast, early treatment with GLV-1h68 resulted in the absence of detectable lumbar or renal lymph node metastases in 7 out of 8 treated mice. Only one out of 8 mice in this group had a slightly enlarged lumbar lymph node. Although all mice in the late treatment group had detectable lumbar and renal lymph node metastases, these metastases were smaller in size compared to the PBS group. LN, lumbar lymph node metastases; RN, renal lymph node metastases.
(TIF)

Table S1 **Capture efficiency, detection efficiency, infection efficiency and infection specificity of the VACV-Cytospin assay with both human and mouse blood samples.**
(DOCX)

Table S2 **Clinical information of 7 patients with metastatic breast cancer.**
(DOCX)

Table S3 **Clinical information of patients with metastatic rectal cancer, lung cancer and melanoma.**
(DOCX)

Table S4 **Clinical information of the patients enrolled in the comparison study.**
(DOCX)

Table S5 **Clinical information of 7 patients enrolled in the CSF study.**
(DOCX)
