# Supplementary figures and images for: Optical Detection and Virotherapy of Live Metastatic Tumor Cells in Body Fluids with Vaccinia Strains
Source: PLoS One. 2013 Sep 3;8(9):e71105. doi: 10.1371/journal.pone.0071105 (PMC3760980; doi:10.1371/journal.pone.0071105)

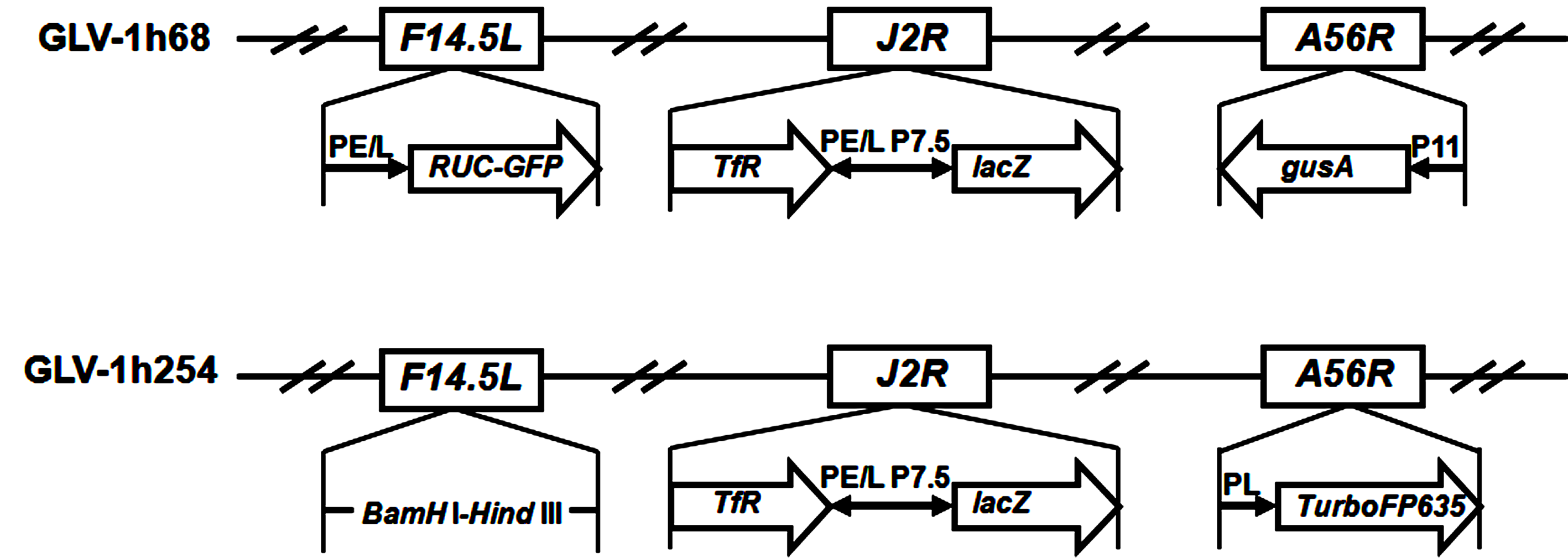

Supplement: Figure S1 — Schematic representation of the genomic structures of the recombinant VACVs GLV-1h68 and GLV-1h254. PE/L, PL, P11, and P7.5 are VACV synthetic early/late, synthetic late, 11K, and 7.5K promoters, respectively. TfR is a human transferin receptor cDNA inserted in the reverse orientation with respect to the promoter PE/L. (TIF) [file pone.0071105.s001.tif]

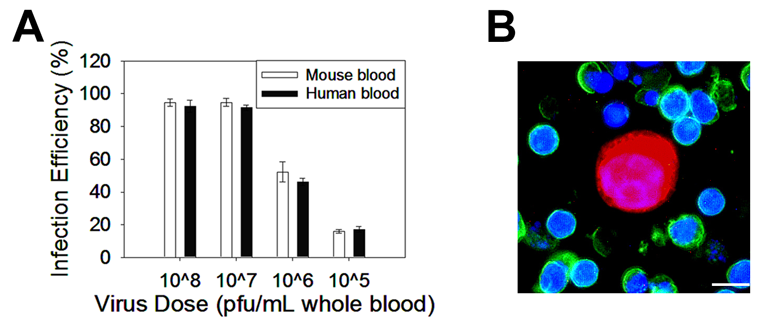

Supplement: Figure S2 — Characterization of VACV-cytospin assay. (A) Infection efficiency of PC-3 cells spiked in the mouse and human blood at escalated doses of virus. (B) GLV-1h254 infected only PC-3 cells spiked in the whole human blood, but not immune cells (red: TurboFP635; green: CD45; blue: nuclei). (TIF) [file pone.0071105.s002.tif]

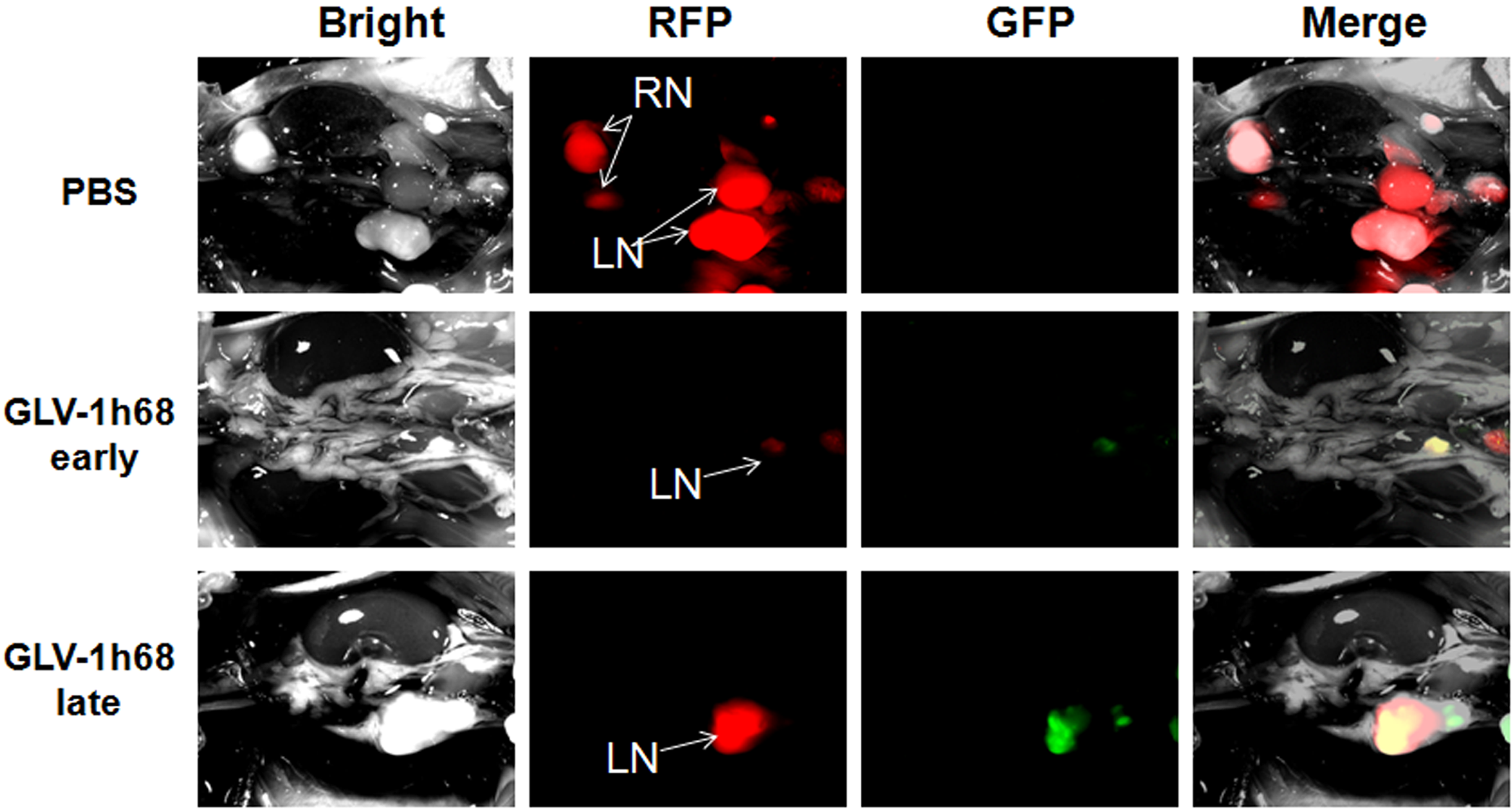

Supplement: Figure S3 — The effect of GLV-1h68 on lymphatic metastases in mice bearing human PC3 prostate cancer xenografts. All mice in the PBS group had detectable lumbar and renal lymph node metastases. In contrast, early treatment with GLV-1h68 resulted in the absence of detectable lumbar or renal lymph node metastases in 7 out of 8 treated mice. Only one out of 8 mice in this group had a slightly enlarged lumbar lymph node. Although all mice in the late treatment group had detectable lumbar and renal lymph node metastases, these metastases were smaller in size compared to the PBS group. LN, lumbar lymph node metastases; RN, renal lymph node metastases. (TIF) [file pone.0071105.s003.tif]
