# Supplementary material for: Optical Detection and Virotherapy of Live Metastatic Tumor Cells in Body Fluids with Vaccinia Strains
Source: PLoS One. 2013 Sep 3;8(9):e71105. doi: 10.1371/journal.pone.0071105 (PMC3760980; doi:10.1371/journal.pone.0071105)
Supplement: Table S1 — Capture efficiency, detection efficiency, infection efficiency and infection specificity of the VACV-Cytospin assay with both human and mouse blood samples. (DOCX) [file pone.0071105.s004.docx]

**Table S1. Capture efficiency, detection efficiency, infection efficiency and infection specificity of the VACV-Cytospin assay with both human and mouse blood samples.**

| Samples | Capture efficiency | Detection efficiency | Infection efficiency | Infection specificity |
| --- | --- | --- | --- | --- |
| PC-3/human blood | 70.61±3.51% | 65.67±4.57% | 92.97±3.72% | 100% |
| PC-3/mouse blood | 72.69±3.78% | 68.04±4.79% | 93.62±4.70% | 100% |
