# Supplementary material for: Optical Detection and Virotherapy of Live Metastatic Tumor Cells in Body Fluids with Vaccinia Strains
Source: PLoS One. 2013 Sep 3;8(9):e71105. doi: 10.1371/journal.pone.0071105 (PMC3760980; doi:10.1371/journal.pone.0071105)
Supplement: Table S2 — Clinical information of 7 patients with metastatic breast cancer. (DOCX) [file pone.0071105.s005.docx]

**Table S2. Clinical information of 7 patients with metastatic breast cancer.**

| Patient ID | Disease Type | Stage | Histology | Disease Status | Prior Therapies | Date of Blood Draw | Date of Last Chemo |
| --- | --- | --- | --- | --- | --- | --- | --- |
| BC1 | Metastatic Breast Cancer | III | Triple negative | Primary | 1. AC & Taxol  2. XRT | 10/10/2011 | 07/06/2011 |
| BC2 | Metastatic Breast Cancer | N/A | ER+ (100%, strong) and PR+ (strong but focal, <5% positivity). HER2/neu immunostain shows CAP/ASCO 2+ positivity. FISH Her2- (However, low level polysomy was detected (average of 3.5 CEP17 signals per cell) | Recurrence | 1. 12/2000 AC x 4  2. Radiation right axilla, IMRT 50.4 gray  3. Tamoxifen x 2.5 years  4. 11/2005: Femara x 2.5 years  5. Chemotherapy with TCH (x 4 only), followed by one year of Herceptin. Last dose 3/8/07  6. Enrolled on vaccine trial at U of Washington:  (plasmid based DNA vaccine encoding the ICD of Her2 (pNGVL3-hICD) with GM-CSF  7. 5/2008 Faslodex until 9/2008 (5 months)  8. 11/2009-approx 6/2010 Xeloda as a single agent  9. 6/2010-12/2010 Olaparib on clinical trial (single agent)  10. 1/7/2011 Abraxane cycle 1, cycle 4 last dose 4/15/2011  11. 6/24/2011 Eribulin  12. 8/15/11: IT Depocyt + MTX q 2 wks  13. 8/22/11-present: Doxil 40mg q 4 wks | 10/11/2011 | 09/27/2011 |
| BC3 | Metastatic Breast Cancer | IIb | ER+/Her2-/PR + (50%) | Primary | 1. Dose dense AC x4, followed by weekly Taxol x 12  2. Adjuvant Tamoxifen | 10/13/2011 | 05/20/2011 |
| Patient ID | **Disease Type** | **Stage** | **Histology** | **Disease Status** | **Prior Therapies** | **Date of Blood Drawn** | Date of Last Chemo |
| BC4 | Metastatic Breast Cancer to the Brain and Leptomeninges | IV | ER+/PR+/Her2**-** | Primary | 1. 6/14/11-6/28/11- WBXRT  2. 8/1/11 – Startd Femara  3. 8/30/11 – Started XRT to spine mets  4. 10/27/11 – IT Depocyt & MTX | 11/03/2011 | 10/27/2011 |
| BC5 | Metastatic breast cancer to brain, possible leptomeningeal disease | T1b N0 Stage I | Her2+/PR+/ER- | Primary | 1. 2003: right lumpectomy/mastectomy  2. 2003-2008: Arimidex  3. 6/2010: liver, lung, bone mets started Taxol/Herceptin which was switched to herceptin alone in 6/2011.  4. 9/1/11-present:WBRT symptomatic brain mets  5. 9/27/11: started Lapatinib & Xeloda BID | 11/03/2011 | 07/27/2012 |
| BC6 | Metastatic Breast Cancer | IIa (pT2N0Mx) | ER+ (100%) PR+ (17%) and Her2- | Primary | 1. Tamoxifen  2. 6 cycles of abraxane  3. XRT  4. Anastrozle  5. Zometa  6. Capecitabine | 01/17/2012 | 04/25/2012 |
| BC7 | Metastatic Breast Cancer | IIIc (T2N3M0) | Triple negative | Primary | 1. AC  2. Taxol/herceptin  3. Radiation  4. Adjuvant herceptin  5. Arimidex | 02/11/2012 | 02/01/2011 |
