# Supplementary material for: Optical Detection and Virotherapy of Live Metastatic Tumor Cells in Body Fluids with Vaccinia Strains
Source: PLoS One. 2013 Sep 3;8(9):e71105. doi: 10.1371/journal.pone.0071105 (PMC3760980; doi:10.1371/journal.pone.0071105)
Supplement: Table S3 — Clinical information of patients with metastatic rectal cancer, lung cancer and melanoma. (DOCX) [file pone.0071105.s006.docx]

**Table S3. Clinical information of patients with metastatic rectal cancer, lung cancer and melanoma.**

| Patient ID | Disease Type | Stage | Histology | Disease Status | Prior Therapies | Date of Blood Draw | Date of Last Chemo |
| --- | --- | --- | --- | --- | --- | --- | --- |
| CC1 | Metastatic Rectal Cancer | N/A | High expression, TS n/a Invalid result, KRAS wild-type | Primary | 1. 02/11/09: 5-FU, leucovorin, status post 10 cycles of modified FOLFOX plus Avastin, status post Cyberknife, s/p cycle 6 of FOLFIRI + Avastin + Zometa  2. 09/30/11 – Present: IT Depocyt/MTX every 2 weeks | 11/10/2011 | 10/14/2011 |
| LC1 | Probable brain metastases of unknown origin and radiation necrosis | N/A | Positive for pancytokeratin, CK7, TTF1 and a few cells staining for CK5/6. CK20 negative. HMB-45 negative. S100 negative. This is consistent with lung or thyroid primary | Primary | No Tx | 11/01/2011 | N/A |
| MM1 | Metastatic cutaneous melanoma to brain, lung, liver and skin | N/A | N/A | N/A | N/A | 02/21/2012 | 06/24/2011 |
