# Supplementary material for: Optical Detection and Virotherapy of Live Metastatic Tumor Cells in Body Fluids with Vaccinia Strains
Source: PLoS One. 2013 Sep 3;8(9):e71105. doi: 10.1371/journal.pone.0071105 (PMC3760980; doi:10.1371/journal.pone.0071105)
Supplement: Table S4 — Clinical information of the patients enrolled in the comparison study. (DOCX) [file pone.0071105.s007.docx]

**Table S4. Clinical information of the patients enrolled in the comparison study.**

| Patient ID | Disease Type | Stage | Histology | Disease Status | Prior Therapies | Date of Blood Draw | Date of Last Chemo | Comments |
| --- | --- | --- | --- | --- | --- | --- | --- | --- |
| 01 | Metastatic Breast Cancer | IIIb | Adenocarcinoma | Primary | Carbo/taxol/herceptin | 05/10/2012 | 05/30/2012 | Sample drawn prior to chemotherapy |
| 02 | Metastatic Colon Cancer | IV | Adenocarcinoma | Primary | XELOX, Avastin, erbitux, MNT1526a (study drug) + avastin, camptosar/erbitux, vectibix/oxaliplatin | 05/17/2012 | 05/17/2012 | Sample drawn prior to chemotherapy |
| 03 | Metastatic Colon Cancer | IV | Adenocarcinoma | Primary | Xeloda/emend/oxaliplatin, Avastin/camptosar/erbitux/5FU | 05/30/2012 | 05/30/2012 | Sample drawn prior to chemotherapy |
| 04 | Metastatic Breast Cancer | IV | Adenocarcinoma | Primary | Taxotere/adria/cytoxan, carbo/gemzar, xeloda/abraxane/avastin, ixempra/avastin, halaven/xgeva | 06/01/2012 | 06/01/2012 | Sample drawn prior to chemotherapy |
| 05 | Metastatic Breast Cancer | IV | Adenocarcinoma | Primary | Faslodex/ Avastin, Gemzar | 06/14/2012 | 06/14/2012 | Sample drawn after chemotherapy |
| 06 | Metastatic Colon Cancer | IV | Adenocarcinoma | Primary | Xelox + Avastin | 07/10/2012 | 07/10/2012 | Sample drawn after chemotherapy |
| 07 | Metastatic Breast Cancer | IV | Adenocarcinoma | Primary | Ariamycin, CMF, Tamoxifen, Femara, Faslodex, Abraxane | 07/17/2012 | 08/28/2012 | Sample drawn prior to chemotherapy |
| 08 | Metastatic Colon Cancer | IV | Adenocarcinoma | Primary | FOLFOX, FOLFIRI | 07/18/2012 | 07/30/2012 | Sample drawn prior to chemotherapy |
| 09 | Metastatic Colon Cancer | IV | Adenocarcinoma | Primary | FASLODEX, Zometa | 09/06/2012 | 09/06/2012 | Sample drawn prior to chemotherapy |
| 10 | Metastatic Breast Cancer | III | Ductal carcinoma | Primary | Taxotere. Cytoxan, Adriamycin | 10/15/2012 | 10/15/2012 | Sample drawn after chemotherapy |
