# Supplementary material for: Optical Detection and Virotherapy of Live Metastatic Tumor Cells in Body Fluids with Vaccinia Strains
Source: PLoS One. 2013 Sep 3;8(9):e71105. doi: 10.1371/journal.pone.0071105 (PMC3760980; doi:10.1371/journal.pone.0071105)
Supplement: Table S5 — Clinical information of 7 patients enrolled in the CSF study. (DOCX) [file pone.0071105.s008.docx]

**Table S5. Clinical information of 7 patients enrolled in the CSF study.**

| Patient ID | Disease Type | Stage | Histology | Disease Status | Prior Therapies | Date of CSF Draw | Date of Last Chemo |
| --- | --- | --- | --- | --- | --- | --- | --- |
| CSF1 | GBM | WHO IV | PDGFR non-amp, EGFR amp | Primary | 1. Chemoradiation  2. Adjuvant TMZ  3. SRS  4. Nilotinib  5. Avastin + Carbo  6. Zometa IV | 11/29/2011 | 11/29/2011 |
| CSF2 | GBM | WHO IV | N/A | Primary | 1. RTOG 0825 protocol, temodar, radiation and avastin/placebo  2. Adjuvant TMZ  3. Avastin + CPT-11 | 11/30/2011 | 04/26/2011 |
| CSF3 | Metastatic Rectal Cancer | N/A | High expression, TS n/a Invalid result, KRAS wild-type | Primary | 1. 2/11/09: 5-FU, leucovorin, status post 10 cycles of modified FOLFOX plus Avastin, status post Cyberknife, s/p cycle 6 of FOLFIRI + Avastin + Zometa  2. 9/30/11 – Present: IT Depocyt/MTX every 2 weeks | 12/05/2011 | Deceased |
| CSF4 | Metastatic Breast Cancer | IIB | ER+/Her2-/PR + (50%) | Primary | 1. Dose dense AC x4, followed by weekly taxol x 12  2. Tamoxifen  3. IT depocyt, MTX, thiotepa  4. IV Alimta | 05/24/2012 | 08/03/2012 |
| CSF5 | Metastatic Esophageal Cancer | Stage IIIA, T3N1 M0 | EGFR FISH – negative High expression of TOP01, TOPO2A, TOPO2B HER2 amplified High expressin of COX-2, SRC, EPHA2 | Primary | 1. SRS  2. SRS + TMZ  3. CPT-11  4. IT depocyt, MTX, thiotepa  5. Tarceva | 07/11/2012 | 07/25/2012 |
| CSF6 | Metastatic Breast Cancer | Stage IIB, T2N1 | Triple negative | Primary | 1. SRS to brain  2. Herceptin, Taxol, Tykerb  3. Herceptin, Pertuzamab, Taxotere  4. IT depocyt/MTX | 07/16/2012 | 08/01/2012 |
| Patient ID | **Disease Type** | **Stage** | **Histology** | **Disease Status** | **Prior Therapies** | **Date of Blood Drawn** | Date of Last Chemo |
| CSF7 | Metastatic Breast Cancer | N/A | Triple negative | Primary | 1. Topotecan  2. IT chemo w/ depocyt, MTX, thiotepa  3. IV Alimta  4. Whole brain radiation | 07/16/2012 | 07/17/2012 |
